# A comparison of obstetrics and perinatal outcomes of teenagers and older women: Experiences from rural South Africa

**DOI:** 10.4102/phcfm.v2i1.171

**Published:** 2010-11-02

**Authors:** Monjurul Hoque, Shahnaz Hoque

**Affiliations:** 1 Empangeni Hospital, Empangeni, South Africa; 2 Centre for Child and Adolescent Mental Health, University of Bergen, Norway

**Keywords:** low birth-weight delivery rate, pregnancy outcome, preterm delivery rate, stillbirth rate, teenage pregnancy

## Abstract

**Background:**

Teenage pregnancy is a known risk factor for a negative pregnancy outcome and poses a health risk to teenagers; it is thus considered a public health problem. It is also an indicator of problems with the sexual and reproductive health of a country's young population. In South Africa, most of the adolescent pregnancies are to be found within the context of unstable relationships with the father of the baby and are unplanned or unwanted.

**Objectives:**

This study estimates and compares the incidence of adverse obstetric and perinatal outcomes of teenage women with older women, to identify specific health needs of teenage mothers during pregnancy and delivery.

**Methods:**

A retrospective cohort study targeted pregnant women who delivered at Empangeni Hospital from April to December 2005, whilst comparing the obstetric and perinatal outcomes of all teenage (ages < 19 years) pregnant women with those of older pregnant women (ages ≥ 19 years) for this study period. Data were collected from the labour ward delivery registry. Pearson's chi-square test was performed to measure the level of significance (alpha = 0.05) for association amongst variables. The student t-test was used to find the significance difference between two proportions and the binary logistic regression method was employed to find the significant predictor for outcome variables.

**Results:**

There were 7836 deliveries over the study period, of which 1236 (16%) were teenage mothers. The rate of gestational age at delivery (e.g. pre-term delivery of 12%), vaginal and forceps deliveries, foetal presentation at birth, multiple pregnancies, low birth-weight and live births deliveries and mean Apgar scores were similar for both groups. The caesarean delivery rate (20%) and macerated stillbirth rate (1.1%) were significantly lower (*p* < 0.05) for teenagers than for older women.

**Conclusion:**

Although there was a higher rate of teenage pregnancy, it did not appear that it was associated with extra perinatal negative outcome such as preterm delivery, low birth-weight delivery and stillbirth. However, strategies are urgently needed to delay conception and improve the socio-economic development of teenage girls.

## INTRODUCTION

Teenage pregnancy is regarded as a serious public health problem and often occurs in the context of poor social support and maternal well-being.^1^ Maternal and child health programmes in South Africa are located within the framework of general development policies,^2, 3^ which focus on meeting the basic needs of rural and urban communities, maximising human resources potential, enlarging the economy and spreading its benefits, as well as democratising society and its institutions. To comply with these policies, free health care services for pregnant mothers and children under the age of 6 years in public health facilities were introduced in 1998.^2^ Teenage mothers enjoy a similar degree of care to their older peers in public health facilities and, recently, a child support grant was also introduced for mothers.^3^ Teenage pregnancy is known to be associated with adverse pregnancy outcomes, such as preterm births, low birth-weight deliveries, foetal growth retardation and perinatal mortality.^4, 5^ Teenage pregnancy is also linked with the increased risk of assisted delivery and caesarean section,^6^ however, a study has suggested that these effects are related with some other confounders.^7^ A high rate of teenage pregnancy also indicates problems with the sexual and reproductive health of a country's young population, which, again, poses serious implications for other health issues such as the spread of sexually transmitted infections (STIs), including human immunodeficiency virus (HIV). It has been reported that a teenager's first pregnancy results in a greater frequency of adverse perinatal outcomes than the second or subsequent pregnancies.^5, 8^ Studies in the United States of America and the Netherlands have found that younger teenage mothers are more likely to deliver preterm babies than women in their twenties.^9, 10^ These studies have focused on the role of infection and severe psychosocial stress factors – such as social isolation, homelessness and violence – which are more common in teenage mothers worldwide.^11, 12^ It is also evident that preterm deliveries are common in teenagers who experience social trauma such as family violence.^13^

In South Africa, teenage pregnancies are seen to occur within the context of unstable relationships with the father of the baby and are often unplanned or unwanted.^14^ Studies have reported that unplanned pregnancy predisposes women to unsafe abortion and even premature and preventable deaths.^15, 16^ However, the extent of the negative outcome of teenage pregnancy in South Africa is not known, particularly within the rural areas of KwaZulu-Natal Province (KZN). This study was conducted with the objectives of estimating the prevalence of teenage pregnancy and comparing the obstetric and perinatal outcomes of these teenagers with older women who delivered at the rural Empangeni Hospital in KZN over the same study period. This study also attempts to identify the highest risk group for pregnant women in order to improve perinatal outcomes in line with achieving the Millennium Developmental Goals.

## ETHICAL CONSIDERATIONS

Prior permission for utilising hospital data to conduct this study was obtained from the Empangeni Hospital Ethics Committee. No identification of patients or staff was required to present the results.

## METHODS

### Setting and population

Empangeni Hospital is situated in the health district of Uthungulu (one of 11 districts) in KZN and provides maternal and neonatal health services to the residents of the district. According to the 2001 census, the total population of the district is greater than 450 000, of which more than 93% are rural, Black and speak isiZulu, the local language. The district is situated in the eastern and northern part of the province on the Indian Ocean coast and is approximately 170 km north of the province's commercial capital and primary port city of Durban. The district comprises two hospitals and 14 primary health care (PHC) clinics run by the public sector, which serve mainly the rural and poorer people of the district. There are also two private hospitals, predominantly run by private specialists, as well as over 40 general practitioners’ services available mainly in urban areas. The specialists and medical practitioners of this public (Empangeni) hospital visit all clinics to support and train staff and manage complicated maternity cases on a weekly basis.

Antenatal care is provided at all 14 PHC clinics in the district by nurses and midwives, and these clinics cover 70% of the antenatal population in the district. Empangeni Hospital covers only 30% of the antenatal population and almost all (over 95%) of the district deliveries (i.e. deliveries at public health institutions). Antenatal care at all public health facilities in South Africa (including Uthungulu) is provided according to the national protocol and guidelines.^17^ In addition, voluntary counselling and testing for HIV are offered to all pregnant mothers for prevention of mother-to-child transmission of HIV infection. Every mother attending a public health care facility receives a supply of ferrous sulphate (200 mg daily) and folic acid (150 mg daily) for supplementation until the next appointment. To prevent neonatal tetanus, tetanus toxoid immunisation is administered in three doses (0.5 mL intramuscular): the first is given at the booking visit, followed by a second dose 4 weeks later and a third dose 6 months after that. At the first visit, health education on, (1) danger signs and symptoms, (2) self-care in pregnancy, (3) delivery plan and (4) newborn and infant care are given to all pregnant women. Based on the findings of the above examinations and investigations, a final assessment on risk status and a plan for further antenatal care, management of any problems and delivery are made. Once risk factors and/or conditions are identified at any visit, the pregnant mother is referred to the Empangeni Hospital Antenatal Clinic. However, all pregnant women who have received their antenatal care at the public health facilities in this district are admitted to Empangeni Hospital for delivery.

### Study design and data collection

A retrospective comparative study was undertaken that targeted the cohort of teenage pregnant mothers (aged < 19 years) who delivered at Empangeni Hospital from April to December 2005, and compared their obstetric and perinatal outcomes with all other older pregnant mothers at the same hospital (as a control). This period was considered appropriate for the collection of the minimum required sample size for the study, availability of data and researchers’ capacity to collect data. Data were collected from the labour ward delivery register, which was developed and distributed by the National Department of Health and is the only official record of deliveries used at Empangeni Hospital. This register is used to record demographic information (name, age, parity and address of mothers), details relating to pregnancy (gestational age, antenatal care information and complications of pregnancy, e.g. anaemia, where haemoglobin < 10 g), as well as obstetric, labour and perinatal information. The register recorded the following obstetric information: presentation of foetus during labour, plurality, time of delivery (recorded in hours and minutes), mode of delivery (normal vaginal, vaginal delivery using an operative procedure, such as vacuum or forceps, and caesarean section) and complications of delivery (e.g. perineal and/or cervical tear). Perinatal information included were birth weight, birth outcome of babies (live birth, stillbirth, Apgar score in 1 min and 5 min), post-partum bleeding and so on. The attending midwives recorded all the above-mentioned obstetric and perinatal variables. Haemoglobin estimation was done either at week 36 routinely or at the labour ward during admission by using a portable haemoglobinometer. All midwives working at the labour ward were orientated and received in-service training on filling the labour ward register and compilation of weekly and monthly summary presentations at the weekly and monthly perinatal mortality meetings; thus this data source was considered complete and correct.

### Data analysis

The predetermined data to measure specific objectives were entered into Microsoft Excel 2003 and exported to SPSS 12.0.1 for analysis. Frequency tables and cross-tabulations with Pearson chi-square tests were performed to measure the level of significance (5%) for association amongst variables. Standard deviation (SD) and 95% confidence interval (CI) were also used for different rates (where applicable). The student *t*-test was used to find the significant difference between two proportions, and binary logistic regression was carried out to find the significant predictor for the outcome variables. Obstetric outcomes were measured using rates of low birth-weight delivery, preterm delivery, types of deliveries (e.g. vaginal or operative deliveries) per 100 deliveries, foetal presentations (e.g. vertex, breech), multiple pregnancy rates, delivery complications (e.g. third-degree perineal and cervical tear) and Apgar score. The perinatal outcomes were measured using stillbirth (death of the baby in the uterus) and neonatal deaths (death within 7 days of births).

### Definition of terms

All definitions of term used in this study were taken from the Department of Health's *Guidelines for maternity care in South Africa*^17^. As such, ‘preterm delivery’ was considered when mothers delivered babies between week 28 and week 36 of gestational age. ‘Term delivery’ was considered when babies were born between week 37 and week 41 of gestation. Any delivery that occurred at or after week 42 of gestation was considered ‘post-term delivery’.

‘Stillbirth’ referred to birth of a dead foetus weighing more than 1000 g or a dead foetus born after 28 weeks of gestational age. It was conventionally divided into two categories, ante-partum stillbirths, when a foetus died before the onset of labour, which is often referred to as a ‘macerated stillbirth’ (MSB) and intra-partum stillbirths, when foetal death occurred during labour; this is referred to as a ‘fresh stillbirth’ (FSB).

In accordance with the national definition of anaemia in pregnancy, the pregnant women who had haemoglobin level < 10 g/dL were considered as suffering from anaemia.^17^

## RESULTS

There were 7836 deliveries during the study period, of which 1236 (16%) were from teenage girls. Amongst the teenagers, 1156 (93.5%) were primiparous (had not previously been pregnant) compared to 37% in the older women (*p* < 0.05). The mean age of the teenagers was 17.08 years (SD = 1.014 and range 13–18 years), and 25.50 years (SD = 5.289 and range 19–46 years) for the older women, and the difference was statistically significant (*p* < 0.05). Virtually all (> 97%) in both groups had received antenatal care during pregnancy and, on average, made six antenatal visits before delivery (Table 1). The gestational age (preterm, term and post-term) at the time of delivery between the groups was not different. There was no significant difference in the prevalence of anaemia at the time of delivery for the teenage group (12.4%) or older group (13.7%).

**TABLE 1:** Information on pregnancy, labour and delivery of teenage and older mothers who delivered at Empangeni Hospital during April–December 2004

Variables	Percent		*p*-value

	Teenagers (*n* = 1236)	Older mothers (*n =* 6600)	
**Mean Age †**	17.08 (SD = 1.014)	25.5 (SD = 5.289)	*p* = 0.001
Antenatal booking	98.0	97.0	*p* = 0.057
Mean antenatal visits	6.0	6.0	*p* = 0.576
Prevalence of anaemia	12.4	13.7	*p* = 0.213
			
**Parity**			
Nil	93.2	37.0	*p* = 0.001
One or more	6.5	63.0	*p* = 0.001
Multiple pregnancies	1.5	2.3	*p* = 0.091
			
**Foetal presentation at labour**			
Vertex	95.8	94.6	*p* = 0.081
Breech	2.8	3.4	*p* = 0.310
Other	0.2	0.4	*p* = 0.209

† SD, standard deviation.

Values are given as means (*p* = 0.0).

Foetal presentations (e.g. vertex, breech), multiple pregnancy rates, delivery complications (e.g. third-degree perineal and cervical tear) at the time of delivery were similar in both groups. A significantly higher rate (*p* < 0.05) of episiotomy (44%) was given to teenage mothers during delivery compared to older mothers (20.4%). The caesarean delivery rate was the second highest mode of delivery in both groups, but was significantly higher (*p* < 0.05) in older women (26%) compared to teenage mothers (20%). The assisted delivery (vacuum delivery) rate was significantly higher (*p* = 0.001) in teenage mothers (4.5%) compared to older mothers (2.7%).

Although the mean birth weights of the babies for the groups were significantly different, the low birth-weight delivery rates were similar at 14.3% and 13.7%, respectively, for teenagers and older mothers (*p* > 0.05). There was no difference in the FSB rate between the groups, but a significantly lower (*p* < 0.05) rate of MSB was observed for the teenagers (1.1%) than for the older women (2.1%). Similarly, the Apgar scores of babies at 1 min and 5 min were similar for those born to teenagers and older mothers (Table 2).

**TABLE 2:** Obstetric and perinatal outcomes of teenage and older mothers

Variables	Percent		*p*-value

	Adolescents	Older mothers	
**Gestational age at delivery**			
Preterm delivery	12.1	12.2	0.952
Term delivery	85.7	86.0	0.766
Post-term delivery	1.6	1.8	0.651
			
**Mode of delivery**			
Normal (vaginal)	74.9	71.2	0.057
Vacuum	4.4	2.7	0.001
Forceps	0.7	0.3	0.063
Caesarean	20	25.8	0.001
Episiotomy required	43.6	20.4	0.001
Third-degree perineal tear	0.1	0.3	0.166
			
**Birth weight**			
Mean birth weight	2918 g	2998 g	0.001
Low birth-weight rate	14.3	13.7	0.560
			
**Perinatal outcomes†**			
Live birth	97.5	96.7	0.145
FSB	1.1	1.2	0.848
MSB	1.1	2.1	0.023
			
**Mean Apgar score**			
In 1 min	7.97	8.03	0.846
In 5 min	9.48	9.44	0.923

† MSB, macerated stillbirth; FSB, fresh stillbirth.

Values are given as means (*p* = 0.0).

Applying the Chi-square test of association, we found that multiple pregnancy (*p* = 0.001), breech presentation (*p* = 0.001) and stillbirths (*p* = 0.001) were statistically associated with the low birth-weight of infants. The occurrences of FSB and MSB were significantly associated with the parity of the pregnant women (*p* = 0.001), breech presentation at delivery (*p* = 0.001), forceps delivery (*p* = 0.002) and low birth-weight (*p* = 0.001) deliveries. Applying binary logistic regression, the study did not find teenage pregnancy as a significant predictor for negative pregnancy outcomes, such as preterm delivery, low birth weight and MSB (Table 3).

**TABLE 3:** Results of the logistic regression output for pregnancy outcome variables

Dependent variables	Regression coefficient	*p*- value	OR	95.0% CI for OR	

				Lower	Upper
Caesarean delivery	-0.201	0.066	0.818	0.660	1.013
Macerated stillbirth	-0.939	0.007	0.391	0.197	0.778
Low birth weight	-0.019	0.897	0.981	0.738	1.304
**Gestational age**					
Preterm delivery	0.125	0.721	1.133	0.571	2.247
Term delivery	0.004	0.991	1.004	0.536	1.879
Constant	-2.229	0.000	0.108	-	-

## DISCUSSION

The rate of teenage pregnancy (16%) in this community is higher compared with the national rate of 12% (estimated from the *South African demographic and health surveys 2003*^18^), and with the data from developed countries, for example, Sweden (0.7%), France (0.9%) Canada (2%) and USA (4.9%).^19^ This could have been due to change in teenagers’ sexual behaviours and activities. It has been observed that girls of younger ages in South Africa are involved in sexual activities. A similar rate of teenage pregnancy was also observed from another district of KZN.^20^ A study has shown that because teenagers frequently have sex without reliable contraceptive protection and are often the victims of forced sexual initiation, there is a high rate of teenage pregnancy.^21^ Conditions and behaviours that produce high levels of teenage pregnancy are also likely to bear upon the risk of acquiring HIV. The need to reduce the risk of teenage pregnancy has thus warranted an urgent strategy to be introduced because of the concomitant risk of contracting HIV.

There was no significant difference between the prevalence of anaemia in both groups. Iron deficiency anaemia is the most well known prevalent nutritional deficiency problem affecting pregnant women.^22^ Iron deficiencies develop during pregnancy because of the increased iron requirements to supply the expanding blood volume of the mother and the rapidly growing foetus and placenta.^23^ Every mother attending a public health care facility received a supply of ferrous sulphate (200 mg daily) and folic acid (150 mg daily) as supplementation (prophylaxis), from the first antenatal visit. This was part of standard management of all pregnant women during the antenatal period.^17^ Haemodilution is considered an important physiological adaptation to normal pregnancy. Women who do not haemodilute have higher incidence of pre-eclampsia, although the prevalence of this condition was found to be lower in other studies.^24, 25^ It is therefore not unlikely that teenage mothers could adapt and fulfil the requirements of iron deficiency during pregnancy.

This study has demonstrated that the obstetric risks of pregnancy in teenage women are generally at the same level as that of older women, except for a higher rate of assisted delivery (e.g. use of vacuum and episiotomy). A probable reason for this could be the poor maternal effort to push the baby out during delivery. It is also logical that teenage mothers would be at different stages of their physical strength and immaturity, and lack previous experience of delivery. On the other hand, assistance to teenage mothers during delivery by episiotomy is recommended as a routine practice in South Africa,^17^ therefore a higher rate of episiotomy was expected.

The caesarean delivery rate was significantly lower amongst teenagers, a finding that contrasts with other studies.^6, 7, 26^ This lower rate of caesarean delivery was unexpected and one possible explanation could be the reluctance of obstetricians to perform elective caesarean delivery on teenagers, especially those who were unmarried or without a stable relationship. Teenage mothers also want to avoid a scar that would forever remind them of this pregnancy and jeopardise any future reproductive performance. On the other hand, the higher rate of caesarean delivery amongst older women could be due to the indications of performing such procedure in their previous caesarean deliveries and thus a higher rate could be expected. The decision to undergo caesarean section for delivery was made on the basis of obstetric reasons only. The standard protocol in Empangeni Hospital is that pregnant women with two or more previous caesarean deliveries would have elective caesarean delivery for subsequent pregnancies at term (between week 37 and 38 of gestation). However, we did not know the rate of previous caesarean deliveries amongst older women and so this should be considered as a limitation of the study. The main reasons for caesarean delivery (e.g. cephalo-pelvic disproportion, foetal distress, etc.) amongst the groups were not known from this study; however, the conventional belief is that teenage mothers are less mature physically and the size of the pelvis in teenage mothers might be significantly smaller than in the older mothers. One would therefore expect that, given the same foetal size, teenage mothers should have a higher instrumental and caesarean delivery rate, but this is disproved by our findings. It has been found that longitudinal growth can occur in teenagers during pregnancy, the increment being greater in primiparous pregnancies compared with multiparous pregnancies. It is also known that pregnancy enhances the growth of the pelvis in teenagers.^27^

This study was limited to those women who delivered at Empangeni Hospital. In KZN, it is estimated that over 95% of women attend antenatal care facilities.^6, 7^ The exact proportion of pregnant women who deliver at Empangeni Hospital is not known, but it is estimated that over 95% of all health facility deliveries in the district are conducted in Empangeni Hospital because all other PHC facilities are closed after hours, providing antenatal care during the day (08:00–16:00) only. The assessment of pregnancy and obstetric conditions were limited to the data source (i.e. the maternity register). Other obstetric conditions (e.g. eclampsia, pre-eclampsia, ante-partum haemorrhage, etc.) were not available in the register and thus were not evaluated. However, overall perinatal outcomes (e.g. low birth-weight delivery, stillbirths) were assessed in this study. A higher rate of antenatal attendance and higher numbers of average antenatal visits were observed for both groups (teenage and older mothers), as access to healthcare for maternal healthcare was free at the public health facilities. Therefore, one can assume that teenage pregnant mothers attended and received the same standard of care as older pregnant women, but without any prohibiting factors in their attempt to seek care and support. Higher attendance and care thus might have improved overall teenage pregnancy outcome in this population.

We also found that incidences of preterm delivery and low birth-weight delivery rates were similar in both groups, but at a higher rate (12% and 14%, respectively) compared to Brazil (the rate was 9%), a country with the similar level of economy.^28^ We have demonstrated that the obstetric outcome of teenage pregnancies in this hospital or population was similar to older mothers. There was also no difference in the extent of prenatal, delivery and post-natal delivery care. A comparison between teenage and older women could provide a reasonable and clear picture of whether teenage mothers have different obstetric outcomes. A previous study has shown that preterm delivery is more than twice as likely in women with iron deficiency anaemia.^29^ Iron has an important metabolic effect on the function of coenzymes and so the association between anaemia and preterm labour might be caused by iron deficiency rather than anaemia per se. Unlike other studies, we found similar low birth-weight deliveries in teenagers and older mothers.^5, 26, 30^ We found that teenage mothers were not at a greater risk of having a stillbirth than women of older age.

A standard package of services and care for all pregnant women was formulated and implemented by dedicated and trained midwives at PHCs. This is done with the aid of necessary antenatal and delivery service provisions and would have made possible a better pregnancy, obstetric and perinatal outcome for teenage mothers in this population. Our data showed that pregnancy in teenage women was generally associated with small changes in obstetric risk. It is important to emphasise that many confounding variables such as a maternal habit of smoking, drinking alcohol, diabetes, pre-eclampsia, chest or urinary tract infections, were not included in this study.^25^ The age-related obstetric and perinatal risks for teenage women are low and we believe that pregnancy is associated with considerable socio-economic problems, perhaps leading to social exclusion reducing educational, career and economic prospects. The consequences of teenage pregnancy can be detrimental to the health of women and children. Society thus requires paying the cost of teenage pregnancy in welfare support for young mothers caught in a poverty trap.

Given appropriate antenatal care, the pregnancy outcome of teenage mothers was comparable to that of older women. The results of this study highlighted that teenage pregnancy does not carry any extra risk of obstetric and pregnancy outcome, such as low birth weight babies, preterm deliveries and stillbirths, but socio-economic problems may still exist. Thus, strategies are urgently needed to delay conception and improve the socio-economic development of this group.
